# Mesenchymal stem cells lineage and their role in disease development

**DOI:** 10.1186/s10020-024-00967-9

**Published:** 2024-11-11

**Authors:** Qi Xu, Wenrun Hou, Baorui Zhao, Peixin Fan, Sheng Wang, Lei Wang, Jinfang Gao

**Affiliations:** 1 Third Hospital of Shanxi Medical University, Shanxi Bethune Hospital, Shanxi Academy of Medical Sciences, Tongji Shanxi Hospital, Taiyuan, 030032 China; 2 Stem cell Translational laboratory, Shanxi Technological Innovation Center for Clinical Diagnosis and Treatment of Immune and Rheumatic Diseases, Shanxi Bethune Hospital, Tongji Shanxi Hospital, Shanxi Academy of Medical Sciences, Third Hospital of Shanxi Medical University, Taiyuan, 030032 China

**Keywords:** Mesenchymal stem cells, Lineage genesis, Differentiation potential, Rheumatoid arthritis, Osteoarthritis

## Abstract

Mesenchymal stem cells (MSCs) are widely dispersed in vivo and are isolated from several tissues, including bone marrow, heart, body fluids, skin, and perinatal tissues. Bone marrow MSCs have a multidirectional differentiation potential, which can be induced to differentiate the medium in a specific direction or by adding specific regulatory factors. MSCs repair damaged tissues through lineage differentiation, and the ex vivo transplantation of bone marrow MSCs can heal injured sites. MSCs have different propensities for lineage differentiation and pathological evolution for different diseases, which are crucial in disease progression. In this study, we describe various lineage analysis methods to explore lineage ontology in vitro and in vivo, elucidate the impact of MSC lineage differentiation on diseases, advance our understanding of the role of MSC differentiation in physiological and pathological states, and explore new targets and ideas associated with disease diagnosis and treatment.

## Introduction

Mesenchymal stem cells (MSCs) are multifunctional adult stem cells that can be derived from various tissues, such as adult adipose tissue, peripheral blood, endometrial polyps, menstrual blood, bone marrow (BM), neonatal placenta, and umbilical cord tissues (Heo et al. 2016). MSCs have the capacity for self-renewal and multidirectional differentiation (osteoblasts, adipocytes, and chondrocytes), and they secrete several chemokines critical for tissue maintenance and repairing injured sites (Fu et al. 2019; Pittenger al. 1999). MSCs can also contribute to organ function and participate in innate and adaptive immune responses through interactions with monocytes, macrophage polarization and promotion of regulatory T-cells, suppression of immune responses and angiogenesis regulation, and myogenesis (Nagaya et al. 2004). The genetic and multidirectional differentiation potentials of MSCs from various tissue sources differ significantly, and MSCs from the same tissue source exhibit different differentiation trends in physiological and pathological states. For instance, they exhibit a pro-inflammatory phenotype in specific inflammatory environments and are crucial in the initiation and advancement of cancers (Weng et al. 2021; Sun et al. 2020).

Cellular lineage is the development of multicellular organisms, beginning with individual cells, through cell division, differentiation into tissues and organs, and ultimately, the formation of organized whole organisms. The entire process clearly defines the chronological order and spatial location of the formation of each cell, tissue, and organ (Schlissel and Li 2020). Consequently, the affinities of these cells passed from generation to generation during development are similar to those of human family lineages, hence the term cellular lineage. Cellular lineage analysis involves investigating the multiple developmental trajectories of specific types of progenitor cells under specific conditions, a process illustrated in complex dendritic branching. Cellular lineage analysis provides insight into the early development of different animal species and the exploration of cellular self-repair processes and disease development. Genealogy plays a causal role in cell fate in embryonic development, as altered cell division patterns can lead to disease onset and progression (Stent 1998). Single-cell RNA sequencing (scRNA-seq) technology has demonstrated that MSCs can originate from complex and heterogeneous populations.

Furthermore, scRNA-seq data reveal and enhance the concept of multiple lineage initiation, indicating that MSCs can simultaneously express early markers of lineage differentiation tendencies in multiple MSC lineages, promoting or inhibiting physiological and pathological activities in disease cases. MSCs, as suppressor cell populations, could be characterized using scRNA-seq techniques to assess lineage differentiation and explore disease mechanisms to define new therapeutic targets. The clinical application of stem cell transplantation has matured, and these cells are potential therapeutic tools to be used after the development of joint, lung, liver, and myocardial pathologies (Lv and Niu 2021). However, MSCs should be used cautiously in vivo since they can lead to multiple differentiation profiles because MSCs are relatively homogeneous in immunomodulation (Freeman et al. 2015).

## Genealogical analysis methods

The stem cell cluster at the blastocyst stage of the embryo eventually generates all the mature terminally differentiated cell types in the organization by regulating their ability to differentiate spectrally, ultimately driving organ formation throughout development (Slack 2008). In adult physiology and pathology, stem cells sustain the ability of tissues and organs to repair and regenerate themselves and are sometimes involved in disease development. For instance, with cancer, a subpopulation of tumor stem cells could escape treatment and regenerate to form a new tumor. Therefore, it is essential to understand the diversity and underlying mechanisms of stem cell lineage differentiation under various physiological and pathological conditions. Tracing cellular lineages is currently a pivotal analytical tool and is the gold standard for inferring the relationship between an ancestor and its offspring. The development of single-cell sequencing technologies and novel imaging techniques has recently led to innovative approaches and the analysis of cellular genealogy.

### Traditional genealogical tracing in individual development

Cell lineage tracing is a method for identifying all the offspring produced by a single cell or the same group of cells, pioneered by Charles Whitman in the 1870s in nematodes (Stent 1998). Cell lineage tracing techniques are vital for illuminating the molecular mechanisms behind several fundamental biological processes. Therefore, the creation of animal models in which cell lineages can be traced in vivo has been a long-term goal of investigation in biology. We can analyze lineage segregation at different time intervals by labeling progenitor cells at different developmental stages and examining their location and marker expression at subsequent time points. Lineage tracing techniques can also reveal the mechanisms of stem and progenitor cell roles in individual development, tissue regeneration, cellular repair, and dysregulation of proliferation and differentiation during tumor formation (Blanpain and Simons 2013) (Table 1).

**Table 1:** Different lineage tracing techniques

Cell type and localization		example		Merits	Limitations
Embryo		Embryo transplantations	Embryo natural body color [Spemann and Mangold 1924]	Intuitive graphics and simple operation	Nonclonal, dilution, accessibility to cells
		Chemical dye	CM-Dil [Ben - Yair et al. 2003]		
		Radioactive dye	Tritiated thymidine [Kaplan and Hinds 1977]		
A group of cells or a single cell	Cytoplasm	DNA, RNA microinjection	pCAGGS-IRES-GFP [Ben - Yair et al. 2011]	Clonal, targeted, amplification of the marker	
		Plasmid	H2B-GFP [Kato et al. 2012]		Dilution, invasive, accessibility to cells
		Nanoparticles	PLGANps [Rescignano et al. 2013]	Targeted, amplification of the marker	
	Nucleus	Transposon-mediated	Tol2 transposon [Tryon et al. 2011]	Permanent expression, targeted, high resolution	Possible toxicity at promoter sites
		Lentiviral-mediated	LV-TSTA-EGFP [Bougioukli et al. 2021]		
		Recombinase	Cre recombinases [Liu et al. 2020]		
	Molecular level	SCLT	Integration barcodes [Chen et al. 2022]	Permanent expression, higher resolution	Unstable barcode expression or potential developmental defects

SCLT: Single-cell lineage tracing technology

Lineage tracing can be categorized into two broad groups: prospective and retrospective tracing (Buckingham and Meilhac 2011). Prospective tracing methods typically use genetic means to label subpopulations of progenitor cells that express specific marker genes and test their differentiated progeny at a subsequent time point. In early stem cell research in mammalian tissues, cell lineage tracing using dye and radiotracer labeling was limited to a small fraction of cells (Kretzschmar et al. 2012). Retrospective tracing methods document the accrual of natural genetic markers in the progeny following cell division, which are used to infer lineage relationships.

**Fig. 1:**
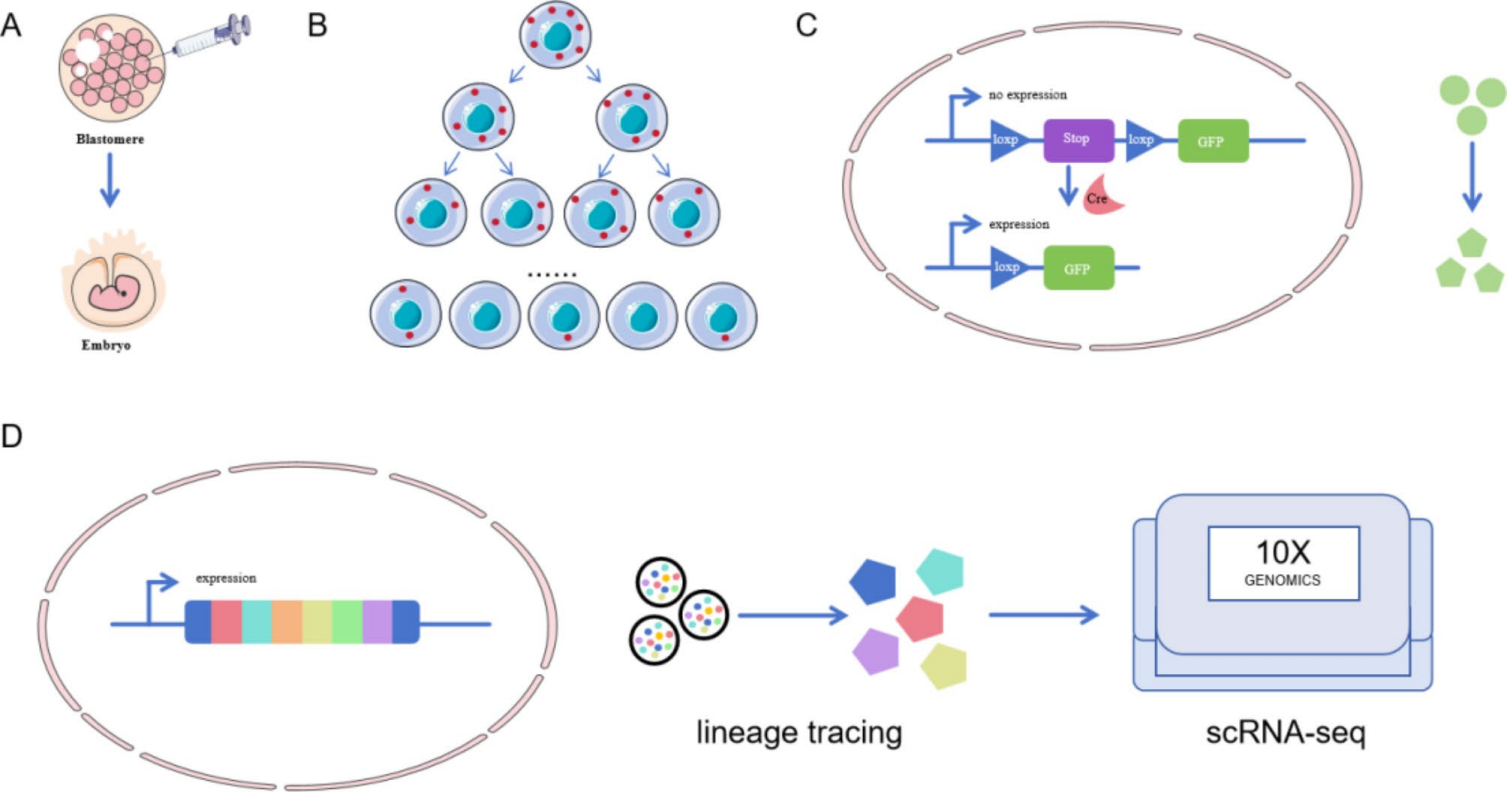
Lineage tracking diagram. (**A**) Labelling of cell clusters with chemical dyes and genealogical tracing to embryonic cells; (**B**) Lineage tracking by markers in the cytoplasm; (**C**) Lineage tracking by markers in the nucleus; (**D**) Single-cell lineage tracing

At the beginning of the 20th century, Spemann, as well as Hilde Mangold, used amphibian animals of closely related species but with different natural colors of embryos for embryo induction and transfer to probe embryonic cell fates (Spemann and Mangold 1924) (Fig. 1). Furthermore, Walther Vogt, who began to track the fate of embryonic cells using dyes in the early 1920s (Vogt 1929). Since then, chemical dyes have been used to directly label cells in amphibian embryos and further genealogically trace them to progeny embryonic cells (Hsu 2015; Ben - Yair et al. 2003). In addition to the embryo’s color and chemical dyes, radioactive dyes have been used to label embryos, such as deuterated thymidine as a DNA probe to track DNA synthesis, which is vital in the study of murine neurodevelopment (Kaplan and Hinds 1977). Tracking cell clumps can help document the formation and evolution of cell lineages in the early stages of embryonic development, revealing the origin and interrelationships of various cell types during embryonic development. However, this technology cannot track the fate of a particular or single cell type.

The advent of microinjection assisted by mechanical pressure or potential difference allowed researchers to label individual cells using chemical dyes. The first to be used were horseradish peroxidase (HRP), which has a high molecular weight and does not diffuse easily between cell membranes, and dextran-conjugated fluorescent dyes (Lawson et al. 1991; Voronov and Panchin 1998). Techniques required to act on the cytoplasm can be achieved by introducing DNA, mRNA, plasmids and nanoparticles into the cytoplasm through microinjection, electroporation, lipid bilayer fusion, or calcium-induced other methods of altering membrane permeability (Ben - Yair et al. 2011; Kato et al. 2012; Rescignano et al. 2013). The genealogical relationships of MSCs have been mapped by introducing genetic markers such as fluorescent proteins (green and red fluorescent proteins) or enzymes (β-galactosidase and alkaline phosphatase) into the cellular level of study (Baron and Oudenaarden 2019). However, using microinjected markers or sequences encoding fluorescent proteins is limited by marker dilutions at each cell division. Therefore, it is only suitable for short-term labeling experiments. It is now more common to stably integrate the sequence-encoding fluorescent proteins into the genome through transgenic or gene targeting to provide permanent cellular markers. Non-targeted approaches include transposons and lentiviruses, such as using Tol2-mediated transposons for lineage tracing in zebrafish to demonstrate that embryonic melanocytes and MSCs are derived from the same bipotent progenitor cells (Tryon et al. 2011) and using LV based two-step transcriptional activation system overexpressing GFP (LV-TSTA-EGFP) vectors to validate the reliability of LV-TSTA vectors for transducing MSCs between humans. This provides a basis for using gene therapy in the bone repair process and the possibility of using gene therapy in isolated skeletal regions during bone repair (Bougioukli et al. 2021).

These two gene recombination-mediated constructs are simple, efficient, and flexible in design. However, the insertion position is random, leading to genomic instability or affecting gene function. In contrast, recombinant nucleases have specific gene recognition sites, which are relatively more specific and stable. Therefore, they occupy a very critical position in cell lineage tracing, such as Cre and Flp site-specific recombinases (Liu et al. 2020). The first experiments used to track genetically induced fate mapping in the progeny of cells expressing Cre recombinase were the Engrailed-Cre line crossed with the β-actin-loxSTOPlox-lacZ line to map the fate of cells that originate from the midbrain-backbrain constriction in mice (Zinyk et al. 1998). Multicolor reporter gene systems for single-cell labeling and clonal analysis have been developed, such as the well-known “Brainbow” (Livet et al. 2007) and “MADM” systems (Zong et al. 2005). In addition to the classical recombinant nucleases mentioned above, other nuclease-mediated genetic tracing for gene editing is also under development. For example, zinc finger nuclease, which were previously used in gene editing, have been used to construct reporter cells constructed with octamer-binding transcription factor 4-EGFP to monitor pluripotency status of human embryonic stem cells (Hockemeyer et al. 2009). However, the specificity of the highly specific meganucleases (He et al. 2014) and TALE nucleases (Zhang et al. 2015) are also envisioned. Therefore, transgenic element promoters and markers for genealogical tracking can be combined on demand and regulated by regulating the rearranged or designed transcription elements.

The emergence of scRNA-Seq has greatly contributed to the development of several fields in developmental and cancer biology. ScRNA-Seq technology relies on factors such as similarity in gene expression profiles or RNA metabolic kinetics to reveal complex and rare cell populations and relationships among them, regulatory relationships among genes, and developmental trajectories of different cells (Zou et al. 2023). In some cases, the probability of generating DNA barcodes carrying the genetic tracer information for gene editing is very low, and detecting thousands of markers and barcodes of thousands of progeny is impossible. Similarly, detecting thousands of markers and progeny is a lot of work. Thus, single-cell lineage tracing technology (SCLT) is formed by the combination of scRNA-Seq technology and barcode-based genetic tracer technology single-cell genealogical tracer technology, which is used to collect information on single-cell transcriptomes and genealogical associations between cells. The diverse barcode classifications allow for the capture of more cellular information, thereby increasing the accuracy and resolution of recognizing cellular genealogical associations (Chen et al. 2022). Caleb et al. developed a lineage and RNA recovery tool that can be recognized using scRNA-seq methods to track changes in the transcriptome of cells with labeled clones over time (Brown et al. 2019).

### Spectrum tracking analysis in disease treatment

The protein expression level and its subcellular localization modulate aspects critical to the differentiation of many cell lines and may be a candidate for therapeutic intervention. MSC transfection with lentiviruses bearing the green and red fluorescent protein resulted in unaffected cell morphology, proliferation, and differentiation, and the stemness of the stem cells was maintained. Fluorescently labeled stem cells are detectable even post-implantation, which offers a unique advantage for stem cell tracer investigations. Fluorescence histochemistry combined with laser confocal microscopy showed that transplanted BM-MSCs could differentiate into hepatocytes, lung epithelial cells, fibroblasts, renal tubular epithelial cells, glomerulocytes, and glomerular vesicle-like membrane cells, which helped to heal injured tissues (Anjos - Afonso et al. 2004). Exogenously labeled fluorescent nanoprobes and red-shifted bioluminescence imaging (BLI) in the near-infrared II region (NIR-II) can substitute the conventional luciferase-based bioluminescence imaging, which enables tissue depth penetration and high spatiotemporal resolution (Jathoul et al. 2014). Compared with conventional analysis, NIR optical imaging analysis of MSCs during disease treatment is preferred. These techniques may be used to monitor the targeted migration, accumulation, proliferation, and differentiation of labeled stem cells at specific sites in post-transplantation mouse models. The distribution, survivability, and dynamics of translocation of vein-transplanted mouse MSCs in the liver in relation to acute liver failure were also visualized and quantified by simultaneous recording of NIR-II and BLI signals from the same mice, elucidating the regenerative mechanisms of mouse MSCs involved in treating acute liver failure (Chen et al. 2018). These imaging findings can be used to predict clinical outcomes and optimize therapeutic regimens. MSCs have previously been induced to generate blood vessels and improve the overall condition of ischemic limbs. However, these cells can produce non-vascular cell types, leading to unstable results (Crisan et al. 2008). Therefore, accurately identifying cell markers to isolate lineage-related subgroups, such as vascular stem/progenitor cells VSPC 1 and VSPC 2, is crucial to further develop MSC-based cell therapies. Zhao et al. greatly help to identify these subgroups by combining multicolor lineage tracking, fluorescent cell sorting, and single-cell sequencing of rainbow mice (Zhao et al. 2023). These help to form functional blood vessels after MSC cell transplantation and inhibit differentiation into other lineages, paving the way for clinical translation.

## Spectral differentiation of MSCs

### In vitro multidirectional differentiation potential

In 2006, the International Society for Cellular Therapy released a basic description of MSCs. The minimal criterion for identifying MSCs is their ability to differentiate into osteoblasts, adipocytes, and chondrocytes. Fundamental research and clinical applications support the critical role of MSCs as the most commonly used stem cell type in the repair of soft tissues (including muscle, fascia, ligaments, tendons, nerve fiber tissue, synovium, and blood vessels) and treatment of hard tissues (including bone and teeth) (Zou et al. 2023). Cloning, recombination, and application of an increasing number of cytokines and increasingly sophisticated stem cell culture techniques and conditions have led to tremendous advances in in vitro expansion and induced differentiation methods, which have been partially and fully successfully applied in regenerative medicine for clinical cell transplantation (Hu and Li 2018). MSCs are superior to other cell sources for in vitro expansion, and the in vitro lineage differentiation of MSCs depends on two major factors: the right cell source and in vitro conditions. Different tissue sources, cell subpopulations, and cell-embedded microenvironments significantly affect the direction of MSC lineage differentiation (Brown et al. 2019).

MSCs are non-hematopoietic cells that differentiate into multiple lineages in vitro under appropriate conditions. The three embryonic layers developed during early embryonic development include mesoderm (lipid-forming cells, osteoblasts, chondrocytes, cardiomyocytes, and vascular endothelial cells), ectoderm (epidermal and neuronal cells), and endoderm (hepatocytes and pancreatic cells), which ultimately differentiate further to produce different tissues and organs.

#### Spectral differentiation into mesodermal cells

MSCs usually differentiate into mesoderm, leading to osteogenesis, adipogenesis, and chondrogenesis. The process of distinguishing MSCs from osteoblasts is a complicated procedure that involves several factors, including inter and intracellular signaling, such as signaling pathways, transcription factors, growth factors, and micro-RNA (miRNA) (Hankenson et al. 2015). Among the epigenetic regulatory mechanisms, histone methylation modification was an essential epigenetic regulatory mechanism during the osteogenic differentiation of BM-MSCs (Zhao et al. 2021). The overexpression of miR-375 significantly enhances the osteogenic effects of human adipose MSCs (hASCs) (Chen et al. 2019), and the proteasome inhibitor bortezomib promotes osteogenesis and inhibits bone resorption in patients with multiple myeloma. BM and synovial-derived MSCs have a higher osteogenic differentiation capacity than periosteal and adipose-derived MSCs.

Furthermore, BM-MSCs can generate an adipocyte lineage through adipogenic differentiation, which converts preadipocytes to mature adipocytes. Accelerated adipogenesis is a vital hallmark of obesity and is critical for developing obesity. For example, peroxisome proliferator-activated receptor-γ could promote lipid metabolism and differentiation of MSCs into adipocytes by regulating cytokine production and influencing the cell cycle (Abbas et al. 2022). Extensive studies have demonstrated that the dysregulation of the adipose-osteogenic balance accompanies the onset and progression of several human diseases, such as obesity, osteosclerosis, and osteoporosis. MSCs can differentiate into different cell lines under the interaction of multiple factors to maintain lipogenic differentiation in a dynamically balanced organism. An in-depth investigation of the mechanism of osteogenic differentiation of MSCs contributes to regulating the adipose-osteogenic balance. It provides significant guidance and new therapeutic ideas for the clinical application of stem cells.

In vitro, the chondrogenic differentiation capacity of MSCs is closely associated with the ability to produce extracellular matrix. Peng et al. found that the tendency for chondrogenic differentiation in the umbilical cord (UC) is higher than that in BM-MSCs and adipose tissue (AT)-MSCs (Wei et al. 2012). BM, synovial, and periosteal-derived MSCs could form matrix clusters with a diameter > 1 mm within 14 days, whereas AT-MSCs have poor chondrogenic differentiation abilities. Sakaguchi showed that the chondrogenic capacity of AT-MSCs was stronger than that of periosteal-separated BM-MSCs. In addition, the loss of chondrocyte homeostasis is an essential pathological alteration (Fujii et al. 2022). The particular characteristics of cartilage tissue, such as the absence of vascularization and lymphatic distribution, cannot adequately perform self-repair. Therefore, artificial repair of cartilage destruction is clinically significant (Jelodari et al. 2022). Insulin-like growth factor-1 (IGF-1) stimulates the proliferation of BM-MSCs while regulating apoptosis and inducing chondrocyte, type II collagen, and SOX9 expression. BM-MSCs and transforming growth factor β (TGF-β) synergistically promote cell proliferation and apoptotic cell death (Longobardi et al. 2009). Recently, with the discovery and in-depth research on stem cells, including their wide range of sources, good proliferative capacity, multidirectional differentiation potential, and good biocompatibility with various biomaterials, stem cells have proven critical in tissue homeostasis and regeneration. This point provides more options and directions for clinical treatment.

MSCs can also be transformed into cardiomyocytes stimulated by specific culture media and inducing factors. In 2004, Xu’s colleagues successfully differentiated human BM-MSCs into cardiomyocytes in vitro (Xu et al. 2004). Subsequent studies have shown that the isolated cardiomyocyte stem cells in vivo have similar biological properties to MSCs, and all of them highly express the surface markers and specific transcription factors of MSCs. This suggests that BM-MSCs can differentiate into cardiomyocytes. The in vitro induction of MSCs differentiation into cardiomyocyte-like cells provides an experimental basis for myocardial regeneration, infarction, and cardiac disease treatment.

MSCs can also differentiate into vascular endothelial cells with specific functions in vivo. The differentiation of vascular endothelial cells, similar to that of cardiomyocytes, usually requires the application of specific culture conditions and inducing factors, such as vascular endothelial growth factors. These induced conditions can contribute to the expression of endothelial cell markers, such as vascular endothelial cell-specific antigen (CD31) and vascular endothelial cell adhesion molecules in MSCs (Jia et al. 2013). These differentiated vascular endothelial cells can be utilized for in vitro vascular engineering and cardiovascular disease research because they are crucial in vascular regeneration and tissue repair. MSCs could be differentiated into cardiomyocytes and vascular endothelial cells in vitro; however, the efficiency of their in vivo differentiation may be influenced by several factors, such as the site of cell injection, local environmental factors, and cell-cell interactions. Therefore, these potential applications will require further research and optimization.

#### Spectral differentiation into ectodermal cells

MSCs can also be induced to differentiate into epidermal and neural cells in vitro, and these cell types can be applied in regenerative medicine and tissue engineering. The differentiation of epidermal cells requires high concentrations of insulin and growth factors such as epidermal growth factor (EGF), keratinocyte growth factor, hepatocyte-like growth factor, and retinoic acid (Polisetty et al. 2008). MSCs express specific biomarkers, such as cytokeratins and cell surface molecule e-calmodulin, during transformation into epidermal cells. In in vitro trauma healing experiments, angiotensin II facilitated the proliferation and migration of MSCs, which induced their differentiation to keratin-forming cells (Jiang et al. 2019). Negative pressure affects cell proliferation and differentiation. Therefore, with increasing pressure, the proliferation ability of MSCs is inhibited, and their ability to differentiate into epidermal cells gradually increases.

MSCs additionally differentiate into neuronal lineage cells such as neurons, astrocytes, and oligodendrocytes in vitro and induce neuronal cell regeneration, cerebral angiogenesis, inhibition of neuroinflammation, maintenance of the integrity of the blood-brain barrier, and degradation of abnormal protein aggregates. The differentiation of MSCs into neurons requires two factors: expression of nested proteins and direct MSC-neuron cell-cell interactions (Wislet - Gendebien et al. 2005). When cultured in a medium containing EGF and basic fibroblast growth factor, MSCs were successfully induced into nestin (+) neurospheres. These neurospheres are differentiated into neurofilament (+) neurons or glial fibrillary acidic protein (+) glial cells, only following further transfer to a neural precursor cell base medium for culture (Kim et al. 2006). During targeted in vitro induction, brain-derived neurotrophic factors promoted the differentiation of MSCs into neuronal cells, producing sufficient numbers of these cells to treat neuronal diseases (Kim et al. 2021).

Therefore, the differentiation of MSCs into epidermal and neural cells critically depends on several factors, such as cell source, culture conditions, and selection of growth factors and inducers. Thus, applying MSC differentiation into epidermal and neural cells requires further validation and development in basic research and clinical practice.

#### Spectral differentiation into endodermal cells

MSCs differentiate into endodermal cells under specified culture conditions. Culturing with a particular media with the addition of appropriate growth factors and signaling molecules promotes the differentiation of cells in specific directions. MSCs are differentiated into hepatocyte-like cells in vitro by adding growth factors, bile acids, and TGF-β (Banas et al. 2007). Differentiated hepatocyte-like cells express several hepatocyte-specific markers and exhibit several biological functions in mature hepatocytes, including albumin expression, urea secretion, cytochrome P450 activation, low-density lipoprotein uptake, and glycogen storage (Okura et al. 2010). Furthermore, miRNAs can directly transform human MSCs into hepatocyte phenotype in vitro (Cui et al. 2012). Therefore, combining the three-dimensional shock perfusion induction system developed by Wang et al. with a hepatogenic medium is a valuable tool for in vitro hepatic tissue engineering using MSCs (Wang et al. 2012). In vitro, MSCs can also be cultured under specific culture conditions by adding appropriate growth factors and signaling molecules, such as IGF-1, insulin, and TGF-β, to induce the differentiation of pancreatic cells. These signaling molecules promote the differentiation of MSCs to pancreatic cells by activating the IGF-1 and TGF-β pathways. Pancreatic markers, such as NK2 Homeobox 2, NK6 Homeobox 1, pancreatic and duodenal Homeobox 1, insulin, and growth inhibitors, were observed in differentiated islet-like cells. Insulin levels also increased daily in the presence of basal glucose levels (Gopurappilly et al. 2013).

#### Differences in the differentiation profile of MSCs from different tissue sources

The ability to induce differentiation into mesodermal lineages in vitro is one of the unique characteristics of biological MSCs. Epigenetic factors drive the lineage differentiation effect in MSCs, and this can increase the likelihood that BM-MSCs will differentiate into osteoblasts and chondrocytes (Yang et al. 2022). AT-MSCs are most likely to differentiate into adipocytes, and synovial membrane (SM)-MSCs tend to differentiate into chondrocytes. The UC-MSCs have extraordinary potential to differentiate into osteoblasts and chondrocytes and even have a differentiation capacity that exceeds that of BM-MSCs (Li et al. 2014). Therefore, each type of MSC can differentiate into a specific cell type in a manner unmatched by other subpopulations, and this needs to be analyzed on a case-by-case basis, as this can affect their therapeutic use and safety.

#### Different subpopulations of MSCs have different differentiation profiles

MSCs show inconsistent efficacy results in clinical trials because of their functional heterogeneity. Studies have shown that the functional heterogeneity of MSCs can be observed in different tissue sources and cell subpopulations within the same tissue source (Rennerfeldt and Vliet 2016). Different subpopulations can be identified according to their biomolecular markers, with the leptin receptor-expressing and CD271 + subpopulation of MSCs playing a dominant role in bone (Zhou et al. 2014) and cartilage formation and repair, respectively (Mifune et al. 2013). Different subpopulations can also be classified based on their biophysical markers, such as cell morphology, and MSCs of various sizes have different spectral differentiation abilities in vitro (Colter et al. 2001). Using the biophysical characteristics of MSC subpopulations and label-free microfluidic cell sorting, the obtained large-diameter MSCs had a greater advantage over other subpopulations in BM repair (Poon et al. 2015).

The different sub-clusters of the MSCs were associated with each other, and scRNA-seq of human BM-MSCs identified three clusters: a CD26 stemness subpopulation, a chemokine-like receptor 1 (CMKLR1) functional subpopulation, and a proliferative subpopulation. The analysis of the genealogical differentiation trajectories revealed that the stemness subcluster was the basal population of the MSCs that gradually differentiated into functional or proliferative subclusters. Compared with the other two clusters, the CMKLR1 functional subpopulation had a greater capacity for immunomodulation and osteogenic differentiation and a lower capacity for adipose differentiation and proliferation (Xie et al. 2022).

#### Influence of environmental factors on the spectral differentiation of MSCs

The microenvironment where cells live can regulate the lineage differentiation and fate of MSCs. For instance, TGF-β3 promotes chondrogenic differentiation, and dexamethasone, antioxidants, and β-glycerophosphate promote osteogenic differentiation (Langenbach and Handschel 2013). Furthermore, chemically induced differentiation involves alterations in the physical state of MSCs, including adhesion and alterations in cytoskeletal contractility, which can prevent or even reverse the differentiation of BM-MSCs (Kilian et al. 2010). For example, after using noridazole and cytochalasin, the cytoskeletal structure of human BM-MSCs undergoes significant changes during osteogenic differentiation (Rodríguez et al. 2004).

Therefore, regarding in vitro culture techniques, many platforms, such as microfluidic devices and chip environments, have emerged to overcome the drawbacks of two-dimensional culture. These experimental protocols are gravity-driven, maintain good cell viability and differentiation capacities for MSCs, and expose the cultures to heterogeneous laminar flow for experimental purposes (Tenstad et al. 2010).

### In vivo genealogical differentiation studies

#### Spectral differentiation of MSCs in physiological states

Under physiological conditions, MSCs show pluripotency and are capable of self-renewal. These cells are predominantly found in BM, AT, and other adult tissues and are used to maintain tissue homeostasis and repair damaged tissues. However, their lineage differentiation is not as clear and specific under physiological conditions as in experiments conducted in vitro. MSCs are essential in adolescent bone development. Synchronized tracking of skeletal stem cells in the growth plate using a dual homologous recombinase system revealed a dynamic transformation process of cartilage and Lepr + BM-MSCs during puberty, in which Lepr + BM-MSCs were predominantly derived from the growth plate cartilage (Shu et al. 2021). Kurth et al. used iododeoxyuridine/chlorodeoxyuridine labeling to identify a group of quiescent, slow-circulating, non-hematopoietic, non-endothelial MSC-like stromal cells in a mouse model for traumatic knee surface injury. These cells are present in the lining and sub-lining layers and undergo proliferation and chondrogenic differentiation after injury in vivo (Kurth et al. 2011). MSCs could also differentiate into other cell types, such as muscle cells, neurons, and skin cells; however, these differentiation pathways are relatively rare or controversial under physiological conditions, and further research is required to determine their accuracy and potential.

#### Spectral differentiation of MSCs in cell therapy

**Fig. 2:**
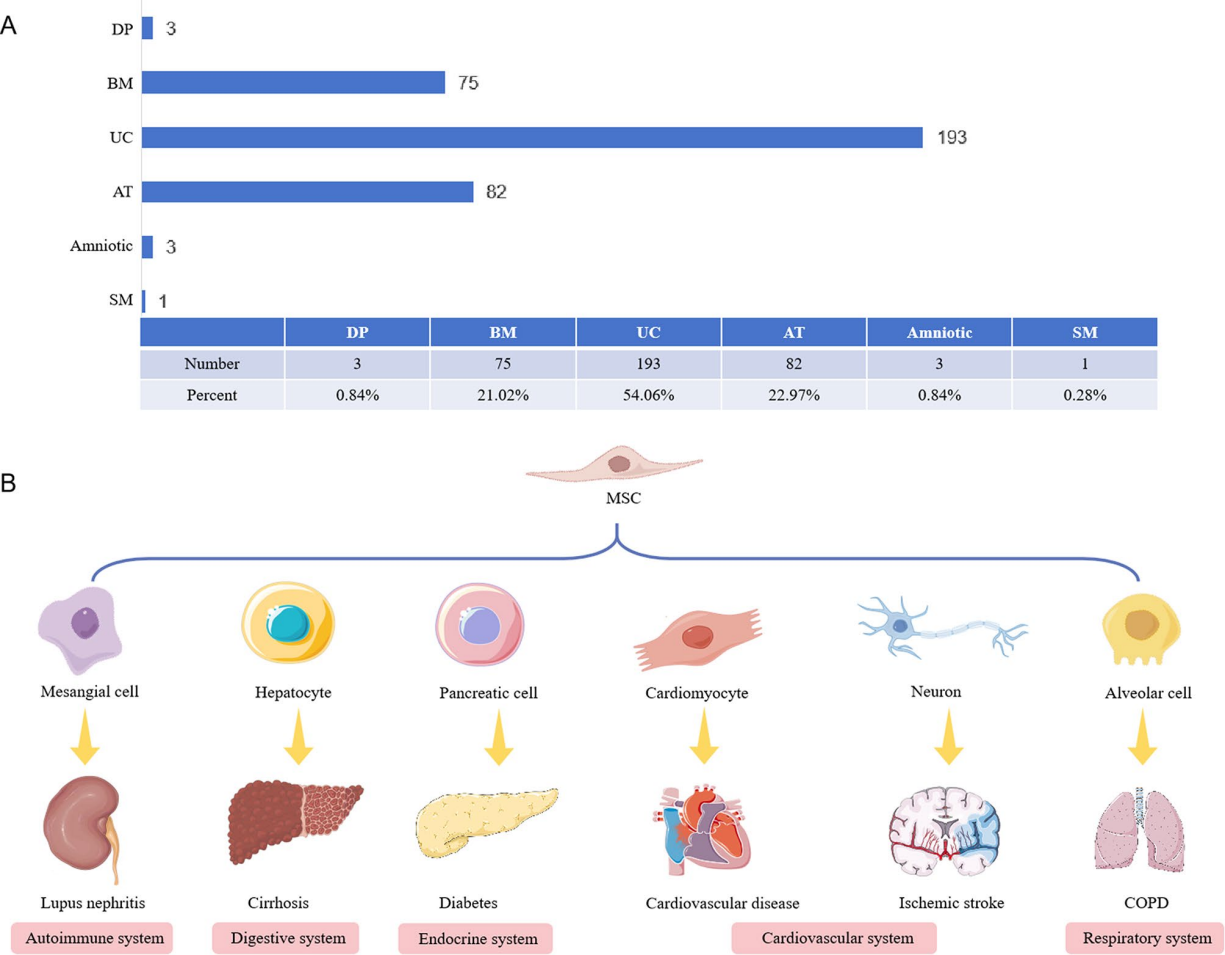
The application of MSCs transplantation in cell therapy. (**A**) Statistical analysis of clinical trials from different tissue sources retrieved on clinicaltrial.gov identifier; (**B**) MSC transplantation and differentiation therapy for various systemic diseases. DP: dental pulp ; BM: bone marrow; UC: umbilical cord; AT: adipose tissue; SM: synovial membrane; COPD: chronic obstructive pulmoriary disease

Cell therapy is a part of regenerative medicine and is one of the crucial medical treatment modalities in recent years. As a representative and promising hotspot of cell therapy, MSC therapy has made desirable progress in many fields due to its strong immunomodulatory ability, use in inflamed and injured tissues, and ease of extraction and isolation. Stem cells are extracted from the body or ex vivo through different methods, such as BM aspiration, AT aspiration, and UC blood collection. The stem cells are cultured ex vivo, the culture environment is regulated, and specific growth factors are added to ensure that the stem cells are guided to differentiate into a specific type of cell, which can then be transplanted back into the patient’s body. MSCs are the most common and safest cells in clinical practice, as they were utilized in > 80% of stem cell therapy programs. Specifically, these programs involve using autologous or allogeneic MSCs for genealogical differentiation or participation in immunomodulation, repair, and reconstruction of organ and tissue function, and modulation of the body’s immune function through intracerebral, pooled, intra-pooled, intranasal, and intravascular routes, such as intravenous or intra-arterial infusions. Genetic differentiation involves immunomodulation, repairing and rebuilding organs and tissue, and modulating immune function. For example, in cell therapy for rheumatoid arthritis (RA), transplanted BM-MSCs can undergo chemotaxis, migration, and colonization of damaged joints and ultimately differentiate into chondrocytes to promote their repair (Gao et al. 2020). BM-MSCs were the first MSCs to be used and have also been extensively studied; however, they are expensive to extract, traumatic to obtain, and extracted in small quantities. Presently, MSC-based genealogical differentiation studies have gradually shifted from bone regeneration to other fields. The applications of MSC transplantation in neurological, cardiovascular, and cerebrovascular diseases have been widely studied and practiced clinically (Fig. 2). AT-MSCs, with their use as a scaffolding material, are expected to be a new strategy to combat atherosclerosis (Yang et al. 2023). In animal models, MSCs express neurons or glial cells in ischemic brain tissue (Wang et al. 2018). Compared with other MSCs, MSCs isolated and cultured from umbilical cords are more primitive and have a stronger proliferative and differentiation ability to differentiate into cardiomyocytes, endothelial cells, and vascular smooth muscle cells in vivo, which is the most promising treatment for cardiovascular diseases (Chen et al. 2021). Moreover, the collection is not harmful to mothers and newborns and complies with medical ethics. Therefore, it is favored by scientists and widely used in clinical research. On the clinicaltrial.gov identifier, 193 clinical studies on UC-MSCs can be retrieved, which is much higher than the studies on MSC from other tissue sources (Fig. 2). The diseases treated are mostly prevalent and difficult to treat at present, including autoimmune diseases (Li et al. 2021), digestive system diseases (Shi et al. 2021), endocrine diseases (Zang et al. 2023), cardiovascular and cerebrovascular diseases (Laskowitz et al. 2024), and respiratory system diseases (Azargoon and Negahdari 2018).

Recently, bioactive scaffolds and scaffolds containing inducible factors have provided improved support for MSC proliferation and differentiation and facilitated the translational application of MSCs clinically. BM-MSCs combined with organic materials (bovine cancellous bone, collagen sponges, polylactic acid, and biomedical porous tantalum), inorganic materials (hydroxylated apatite), and other scaffold materials have been used to significantly promote cellular osteogenesis and bone immunomodulation to facilitate the healing of critical craniomaxillofacial bone defects in mice (Wang et al. 2022).

MSC grafts have multidirectional differentiation potential to promote tissue regeneration and immunomodulatory functions to suppress excessive immune responses and reduce rejection. They also have relatively low ethical controversy and are easily accessible. However, transplantation of allogeneic MSCs may trigger immune rejection, inappropriate differentiation, and formation of abnormally functioning cells or even tumor formation in vivo with prolonged use or high-dose therapy. The lack of human-standardized assays for MSCs to determine metabolism and biodistribution in vivo due to limitations in detection technology and the risk of tumorigenic and tumor-promoting differentiable cells pose great challenges to MSCs in clinical practice and development. Therefore, improving the sensitivity and specificity of these protocols and identifying individualized, efficacious, less toxic, and highly compliant treatment protocols remains a challenge for clinicians and will require further research and exploration.

## MSC genealogical differentiation and disease development

### Joint disease

Arthritis is a chronic condition that affects people of all ages, including children. It is characterized by pain, inflammation, and stiffness in the knees, hips, knuckles, feet, ankles, spine, and shoulders.

**Fig. 3:**
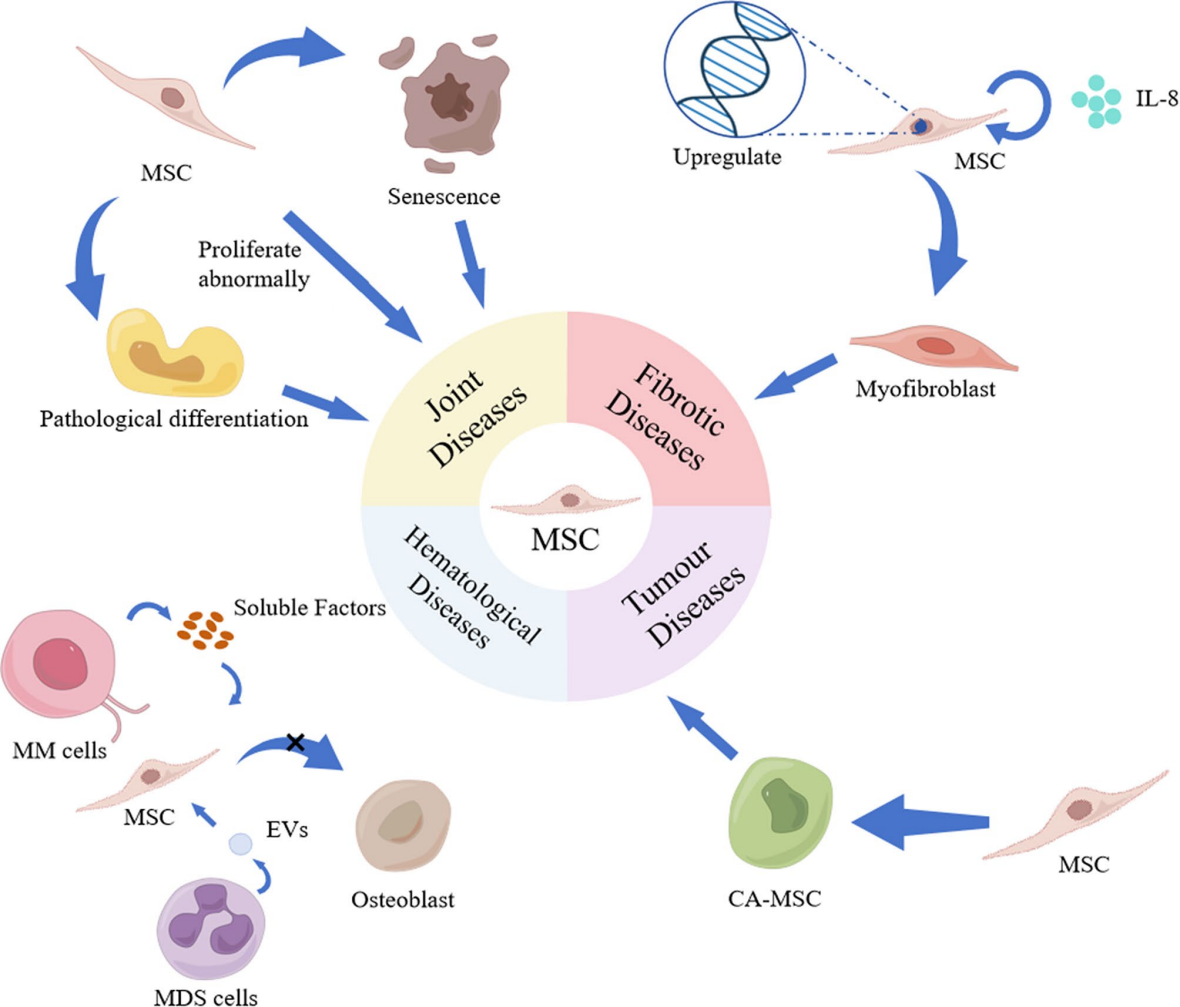
Spectral differentiation of MSCs in disease. Top left: Pathogenesis of MSCs in joint diseases. Top right: Pathogenesis of MSCs in fibrotic disease. Bottom left: Pathogenesis of MSCs in hematological diseases. Bottom right: The pathogenic mechanism of MSCs in tumor diseases. MM cells: Multiple myeloma cells; MDS cells: Myelodysplastic syndrome cells; EVs: Extracellular vesicles; CA-MSC: Carcinoma-associated mesenchymal stem cells

Osteoarthritis (OA) is an age-related progressive degenerative disease that affects various areas around the joints, especially the articular cartilage (Liu et al. 2022). MSCs senescence and exhaustion are significant etiologic factors in OA. Patients with OA receiving arthroplasty have reduced MSCs proliferative activity, chondrogenic and adipogenic activity, and no significant difference in osteogenic activity (Murphy et al. 2002). In addition, MSCs can differentiate into abnormal subchondral bone and blood vessels in the bone of patients with OA. High concentrations of TGF-β in the subchondral bone triggered pathological changes in BM-MSCs in subchondral bone, inducing abnormal bone formation, leading to the development and progression of OA (Zhen et al. 2013) (Fig. 3). MSCs in patients with OA showed upregulated levels of C-X-C motif chemokine receptor 1 (IL-8 receptor) and chemokine receptor 6 (Human macrophage inflammatory protein 3α receptor), which are chemokines that are abundant in the synovial fluid of patients with OA. These chemokines are BM-effective inducers of MSC migration, enabling the MSCs to migrate to specific sites for proliferation and differentiation in tissue repair (Campbell et al. 2016). Therefore, finding and inhibiting the key targets that lead to senescence or pathological changes in MSCs and restoring stem cell performance to a stable state could lead to more effective differentiation into chondrocytes and synoviocytes, thus facilitating the timely replenishment and replacement of senescent joint cells. This approach is safer and more effective than currently available therapies, such as stem cell transplantation.

RA characteristics include persistent synovitis due to the proliferation of synovial endothelial cells, systemic inflammation due to mononuclear cell infiltration, autoantibodies, and massive neovascularization of the synovium (Scott et al. 2010). In the synovial fluid of patients with RA, BM-MSCs were significantly lower compared with those in patients with OA (Jones et al. 2004). In addition, the chondrogenic capacity of synovial MSCs in patients with RA was negatively correlated with the severity of synovitis. These findings suggest that inhibiting the repair function of intra-articular MSCs may be secondary to the accumulation of inflammatory cytokines in patients with RA. The main downstream target of inflammatory cytokines is the transcription factor Nuclear factor-Kappa B (NF-κB), whose sustained activation in fibroblastic synoviocytes (FLS)/MSC cultures is sufficient to inhibit osteogenic and lipogenic differentiation while enhancing proliferation, motility, and matrix-degrading activities (Lee et al. 2007). MSCs are passive targets of the inflammatory process in patients with RA; however, they are also pathogenic. MSCs and FLS can be the same cell type with functional specialization or represent different functional stages of the same matrix spectrum. Arthritic FLSs may be “diseased” MSCs and FLSs and MSCs proliferate and differentiate abnormally in RA joints, transforming through chronic interactions with the inflammatory process of the joints to give rise to more aggressive cell types that can invade the articular cartilage and mediate damage to the articular cartilage and bone (Bari 2015).

AS is a progressive rheumatoid disease characterized by pathologic osteogenesis and ligamentous osteophyte formation, subsequently leading to limited spinal motion and deformity. MSCs are pluripotent stem cells that are the main source of bone formation in vivo (Lv et al. 2014). Patient-derived MSCs have a greater capacity for osteoblastic differentiation than healthy control-derived ones. This point provides a new perspective for inhibiting osteogenic differentiation of pathological MSCs by regulating the balance between bone morphogenetic protein 2 and Noggin (Xie et al. 2016a). Furthermore, analysis of the pathogenic mechanisms at the gene level has identified 16 unique AS single-nucleotide polymorphisms. Single-nucleotide polymorphism adjacent super-enhancer (SASE)-regulated networks regulate the phosphoinositide 3-kinase-protein kinase B, NF-κB, and Hippo signaling pathways by synergistically activating the enhanced osteogenic differentiation of AS-MSCs. The involvement of the SASE-regulated network in the pathological osteogenesis in patients with AS may make it an attractive target for future AS treatments (Yu et al. 2021).

### Fibrotic diseases

Fibrosis can occur in various organs, and the main pathological changes are an increase in fibrous connective tissue and a decrease in parenchymal cells in the organ tissues. This can destroy the organ structure and function, leading to organ failure, which is a serious threat to human health and life. MSCs are often used to treat fibrosis in various organs and tissues because of their ability to repair tissues through “homing” differentiation and immunomodulation; however, they also play an active role in the pathogenic process.

During bone marrow fibrosis (BMF), MSCs experience functional reprogramming during the pre-fibrotic phase and obtain secretory, fibrotic, and osteoblastic phenotypes during marked fibrosis. For example, marker genes are upregulated in myofibroblasts from glioma-associated oncogene homozygous 1+ (Gli1 +) MSCs (MSC-1 upregulates Igfbp7, Limch-1, Wisp-2; MSC-2 upregulates Limch1, Gas7, Acta1) (Leimkühler et al. 2021), as they migrate to the hematopoietic BM to differentiate into stroma-producing myofibroblasts in response to chemokine Cxcl4 secretion by activated platelets, eliciting BMF (Schneider et al. 2017) (Fig. 3). MSCs contained platelet-derived growth factor receptor alpha (PDGFRα) which is necessary for myelofibrosis. Therefore, targeting the PDGFRα pathway in MSCs may be beneficial in treating myelofibrosis. The PDGFRα inhibitor imatinib effectively treated myeloid dysplasia and blocked the expansion, fibrotic transformation, and BMF of BM-MSCs (Decker et al. 2017). Similarly, in MSC-1 and MSC-2, the upregulation of TGF-β, TNF-α, and JAK-STAT signaling pathways is central in patients with BMF (Schneider et al. 2017).

Furthermore, in patients with idiopathic pulmonary fibrosis (IPF), fibrotic mesenchymal progenitor cells autocrine IL-8 promotes the self-renewal of IPF mesenchymal progenitor cells and the proliferation and motility of the IPF mesenchymal progenitor cell differentiated from MSCs. IL-8 also stimulates macrophage migration in a paracrine manner and the infiltration into the adjacent normal alveolar structures, which enlarges fibroblastic foci and contributes to the progression of fibrosis (Yang et al. 2018). MSCs in the lungs expand during fibrosis and form clonal plaques, major contributors to pulmonary fibrosis-associated myofibroblasts, providing new insights into the pathogenesis of progressive fibrotic diseases (Xie et al. 2016b).

ScRNA-seq reveals that MSCs obtained from transcription factor 21 + liver tissues were the major cellular source of hepatic myofibroblasts and tumor-associated fibroblasts. Furthermore, the knockdown of TGF-β receptor type 2, specifically in transcription factor 21 + cells, arrests the progression of liver fibrosis and significantly inhibits the development of hepatocellular carcinoma (Wang et al. 2021). Approximately 35% of renal fibroblasts can be obtained from cells differentiated from BM-MSCs (LeBleu et al. 2013). Genetic lineage tracing has shown that perivascular Gli1 + MSCs proliferate to give rise to myofibroblasts after lung, liver, kidney, or heart injuries. Gli1 + cells comprise a small percentage of the total organ cells and even the PDGFRβ cells; however, their ablation reduces fibrosis in the kidney and heart by approximately 50%. This suggests that Gli1 + MSCs are the main cellular origin for organ fibrosis and are a relevant therapeutic target for preventing solid organ dysfunction after injury (Kramann et al. 2015).

### Diseases of the hematological system

BM is the major hematopoietic organ of the body, and it contains various hematopoietic cells and stroma. Hematopoietic cells include hematopoietic stem cells (HSCs), leukocytes, erythrocytes, and platelets at all growth and developmental stages. The BM stroma is the medium in which the BM cells grow and develop, including MSCs, proteoglycans, and glycoproteins. MSCs are an essential cellular component of the BM microenvironment and are of fundamental and clinical importance for treating poor HSC implantation due to their accessibility to clinical cellular therapies. The evolution of MSC pathology is associated with various hematological disorders and has been identified as a driver of disease development.

Myelodysplastic syndromes (MDS), a group of heterogeneous clonal disorders originating from HSCs, are characterized by abnormal myeloid cell development, leading to hypopoiesis, peripheral hematopoiesis, and a risk of developing acute leukemia (Malcovati et al. 2005). MDS cells impair bone lineage differentiation in MSCs by secreting extracellular vesicles that damage the MSCs and hematopoietic stromal cells generated by the differentiation of the hematopoietic microenvironment, and this negatively affects the ability of the HSCs to inhibit normal hematopoiesis (Hayashi et al. 2022). During the complex pathogenesis of MDS, MSCs have impaired immunoregulatory functions, resulting in a significantly dysregulated immune system (Fattizzo et al. 2020).

Multiple myeloma (MM) is a malignant disease of the plasma cells characterized by the malignant proliferation of monoclonal plasma cells in the BM. Bone disease is one of the major complications of MM that affects the quality of life and survival prognosis; however, 80% of patients with MM have osteolytic lesions, which can affect the differentiation of MSCs to osteoblasts and osteoclast function through the release of soluble factors or cell-to-cell contact. This leads to a decrease in osteoblasts, resulting in impaired bone formation and the development of osteolytic lesions (Toscani et al. 2015). However, increased osteolysis is the only factor affecting the spectrum of myeloma. Heparanase, an enzyme that acts on the cell surface and extracellular matrix by degrading polymerized acetyl heparan sulfate chains, is upregulated in several human cancers, including MM (Mahtouk et al. 2007; Lerner et al. 2008). Heparanase enhances osteoclastogenesis and bone loss and shifts the differentiation potential of MSCs from osteoblastogenesis to adipogenesis (Ruan et al. 2013). Adipocyte-lineage cells can activate Wnt signaling to reduce cleaved cysteine-3 and activate extracellular signal-regulated kinase signaling in MM cells, promoting MM cell growth and chemotaxis (Trotter et al. 2016). Studies have also shown that individuals with obesity are more likely to develop MM than patients with normal weight (Morris and Edwards 2018).

### Tumor diseases

The tumor microenvironment contains several immune cells and other stromal cells, including MSCs, FLS, endothelial cells, and pericytes. MSCs regulate the phenotype and function of all immune cells involved in anti-tumor immunity, and they can also exert an immunosuppressive effect, increasing the rate of tumor metastasis and recurrence (Liang et al. 2021) (Fig. 3).

Cancer-associated fibrosis is an important component of tumor microcircuitry. In addition to fibroblasts and stellate cells, MSCs are crucial in tissue fibrosis and connective proliferation and are essential mediators of fibrosis. Following cancer stimulation, normal MSCs can be transformed into carcinoma-associated mesenchymal stem cells (CA-MSCs). The CA-MSCs are pluripotent cells that can differentiate into tumor microenvironment components, including fibroblasts, myofibroblasts, and adipocytes. Furthermore, the CA-MSCs can express high levels of BMP proteins and promote tumor growth by increasing the number of cancer stem cell-like cells compared with normal MSCs (Coffman et al. 2016). Moreover, the direct contact co-culture of CA-MSCs and ovarian cancer cells can increase ovarian cancer cell adhesion, migration, invasion, proliferation, and chemoresistance, spreading to mesothelial cells and leading to peritoneal metastasis (Lis et al. 2014).

## Discussion

The expansion of lineage analysis has recently resulted in various major scientific breakthroughs. Using technologies such as single-cell sequencing and fluorescent labeling, scientists can trace the differentiation pathways of MSC, thereby gaining insight into their differentiation mechanisms. The development of these techniques has greatly contributed to the progress of MSC lineage studies. In vitro and in vivo research have demonstrated that the source, subpopulation, and environmental factors of MSCs could affect the direction of lineage differentiation, thereby promoting or inhibiting physiological and pathological activities in various diseases. However, there are limitations to the current research on MSCs. For example, genealogical analysis techniques are limited, and most studies analyze MSCs in vitro, whereas research in vivo focuses on animal studies. There are also relatively few investigations into the mechanisms of disease development that are directly associated with human MSCs, including the immune rejection triggered by allogeneic MSC transplantation, the correlation between the pathological transformation of MSCs and their environment, the presence of a potential risk of tumor formation, and the roles occupied by MSCs in various diseases. With continuous progress in differentiation technology, gene modification, clinical application, and multi-omics research, the application of MSC will become more extensive and precise in the future. Notably, more large-scale, multicenter clinical trials will help to tailor MSC treatment protocols to patients, combining genetic analysis and individualized treatment strategies to improve the safety and efficacy of treatment. However, some technical and clinical challenges, such as immune rejection, tumor formation, and heterogeneity, still need to be overcome. MSC technology will bring breakthroughs in regenerative medicine and personalized therapy through multidisciplinary collaboration and innovation.

## Data Availability

Not applicable.

## Abbreviations

MSCs: Mesenchymal stem cells
BM: Bone marrow
scRNA-seq: Single-cell RNA sequencing
HRP: Horseradish peroxidase
LV-TSTA-EGFP: LV based two-step transcriptional activation system overexpressing GFP
SCLT: Single-cell lineage tracing technology
BLI: Bioluminescence imaging
NIR-II: Near-infrared II region
hASCs: Human adipose-derived mesenchymal stem cells
PPAR-γ: Peroxisome proliferator-activated receptorγ
UC: Umbilical cord
AT: Adipose tissue
EGF: Insulin-like growth factor-1
IGF-1: Epidermal growth factor
TGF-β: Transforming growth factor-β
SM: Synovial membrane
CMKLR1: Chemokine-like receptor 1
RA: Rheumatoid arthritis
COPD: Chronic obstructive pulmoriary disease
OA: Osteoarthritis
CA-MSCs: Carcinoma-associated mesenchymal stem cells
FLS: Fibroblast-like synoviocytes
AS: Ankylosing spondylitis
SASE: Single-nucleotide polymorphism adjacent super enhancers
BMF: Bone marrow fibrosis
PDGFRα: Platelet-derived growth factor receptor alpha
IPF: Idiopathic pulmonary fibrosis
Gli1 +: Glioma-associated oncogene homolog 1 +
HSCs: Hematopoietic stem cells
MM: Multiple myeloma
MDS: Myelodysplastic syndromes
NIR-II: Near-infrared II region
